# Reverse End-to-Side Triceps to Axillary Nerve Transfer for Treatment of Axillary Nerve Palsy After Reverse Total Shoulder Arthroplasty

**DOI:** 10.7759/cureus.101108

**Published:** 2026-01-08

**Authors:** Mitchell S Mologne, Michele Christy, Zachary D Randall, Christopher J Dy

**Affiliations:** 1 Orthopaedic Surgery, Washington University School of Medicine, Saint Louis, USA

**Keywords:** axillary nerve injury, axillary nerve transfer, peripheral nerve, reverse end to side nerve transfer, reverse shoulder arthroplasty

## Abstract

Nerve injuries are uncommon, yet significant complications following reverse total shoulder arthroplasty (rTSA), with the axillary nerve being most frequently injured. We report a case of a 67‐year‐old female who developed persistent motor dysfunction and decreased sensation in the axillary distribution following rTSA for a comminuted proximal humerus fracture. Electrodiagnostic studies confirmed right axillary nerve injury with reduced motor unit recruitment. After no improvement in deltoid function at three months following rTSA, we discussed treatment options for the patient, who opted for surgical intervention. Based on intraoperative findings, we performed a reverse end‐to‐side triceps‐to‐axillary nerve transfer. Postoperative evaluations over a four‐year follow‐up revealed substantial improvements in range of motion, strength, and patient‐reported outcomes. Ultrasound and magnetic resonance imaging studies obtained at four years post-op demonstrated mild deltoid atrophy and fatty infiltration, despite the excellent clinical result. The triceps‐to‐axillary nerve transfer has potential as a viable intervention for non-recovering axillary nerve injuries after rTSA.

## Introduction

Nerve injuries, though rare, are significant complications following reverse total shoulder arthroplasty (rTSA), affecting up to 1.3% of patients [1]. The axillary nerve is most commonly involved, particularly in cases performed to treat proximal humerus fractures [1]. These nerve injuries may often resolve completely with conservative management; however, some patients may continue to experience persistent pain and dysfunction [2-3]. Typically, these nerve palsies recover in 6-7 months with early signs of clinical recovery; however, prognosis is much poorer when there are little to no signs of clinical recovery at 3-4 months [2, 4-7]. In these challenging situations in which an acute denervation occurred but there is poor clinical recovery and/or signs of delayed reinnervation, surgical exploration with quadrangular space decompression and intraoperative nerve testing to guide further surgical decision making is indicated [5, 7].

Prior reports have described the use of a triceps-to-axillary nerve transfer to treat axillary nerve injury prior to rTSA. However, we describe a 67-year-old female who underwent treatment of a presumed iatrogenic axillary nerve injury identified after rTSA for treatment of a proximal humerus fracture. Given the patient’s disability, preference for definitive treatment, the reliance on sufficient deltoid function for optimal rTSA mechanics, and her poor motor recovery on nerve testing, the patient opted to undergo surgical intervention. After the quadrangular space decompression and intraoperative nerve conduction testing was conducted, it was decided to proceed with a reverse end-to-side nerve transfer to treat her nerve palsy [5]. Given the increasing utilization of rTSA to treat proximal humerus fractures and the risk of axillary nerve injury, this case may provide insight into treatment options for patients with delayed clinical recovery of their nerve palsy [8].

## Case presentation

Written and verbal consent from the patient was obtained to share this case.

Our patient is a 67-year-old right-hand dominant female with no significant past medical/surgical history. She sustained a comminuted, 4-part proximal humerus fracture while skiing and underwent an acute right rTSA for fracture 10 days later at an outside hospital. In the emergency room post-accident, the patient was documented to have an intact neurovascular exam with specifically noted normal sensation in an axillary nerve distribution, with the patient also denying numbness, tingling, or weakness; however, the range of motion and motor function of the shoulder were unable to be assessed due to pain. After surgery, she experienced decreased sensation over the lateral aspect of her right shoulder and weakness in shoulder flexion, abduction, and extension. Nerve conduction studies performed three months later revealed right axillary focal neuropathy with significantly decreased compound muscle action potential and a reduced recruitment pattern with only 2-3 motor units present (Table 1).

**Table 1:** Electrodiagnostic Studies of the Axillary Nerve MUAPs: Motor Unit Action Potentials; Ins Act: Insertional Activity; Fibs: Fibrillations; Pos Waves: Positive Sharp Waves; Fasc: Fasciculations; Amp: Amplitude; Dur: Duration; Config: Configuration; Poly: Polyphasic Potentials; Interf Pattern: Interference Pattern; Inc: Increased; Norm: Normal; Fr Decr: Frequently Decreased; Gr Decr: Greatly Decreased

	Insertional	Spontaneous Activity			Volitional MUAPs					Maximal Patient Effort		
	Ins Act	Fibs	Pos Waves	Fasc	Amp	Dur	Config	Poly	Pattern	Recruitment	Interf Pattern	Effort
Deltoid	Inc	4+	4+	None	Inc	Inc	Norm	Few	Fr Decr	Gr Decr	2-3 units	Full
Infraspinatus	Norm	None	None	None	Norm	Norm	Norm	None	Norm	Norm	Reduced	Full
Supraspinatus	Norm	None	None	None	Norm	Norm	Norm	None	Norm	Norm	Reduced	Full
Biceps	Norm	None	None	None	Norm	Norm	Norm	None	Norm	Norm	Norm	Full
Triceps	Norm	None	None	None	Norm	Norm	Norm	None	Norm	Norm	Norm	Full

Given the findings of her electrodiagnostic studies, she was referred to our clinic one month after the nerve studies (4 months after her initial surgery). Physical exam on presentation showed decreased sensation in the axillary cutaneous distribution and decreased range of shoulder motion, with 60° of forward flexion, 20° of composite shoulder abduction, and 30° of extension. There was no palpable anterior middle deltoid function when isolating on examination, but there was some contraction of her posterior deltoid. Her initial patient-reported outcomes can be seen in Table 2.

**Table 2:** Pre- and Postoperative Patient-Reported Outcomes PROMIS: Patient-Reported Outcomes Measurement Information System

Patient-Reported Outcome	Pre-Nerve Transfer	4 Years Post-Nerve Transfer	Difference
PROMIS Upper Extremity	26.3	40.7	14.4
PROMIS Pain	61.5	47.8	-13.7
PROMIS Physical Function	32.7	50.2	17.5

Given the need for optimal deltoid function with an rTSA, a lengthy discussion of both operative and nonoperative treatment options was conducted with the patient. It was mentioned to the patient that there was a possibility that the nerve injury would spontaneously heal on its own; however, given her degree of disability, lack of noticeable improvement, and her desire for definitive treatment, she ultimately decided to undergo surgical intervention. The plan was to stimulate the axillary nerve intraoperatively to guide the need for continued observation, end-to-end nerve transfer, or reverse end-to-side nerve transfer.

Nerve transfer operation

Our patient underwent a nerve transfer of the right long head of triceps to the anterior branch of the axillary nerve in a reversed end-to-side fashion. A posterior approach was taken to the quadrangular space and triangular interval. A skin incision was made along the posterior aspect of the upper brachium. Dissection was performed to the level of the posterior deltoid and triceps interval, where the cutaneous branch of the axillary nerve was identified and used as a guide to enter the quadrangular space. The axillary nerve was decompressed, releasing a portion of the teres major, which allowed for identification of the posterior branch of the axillary nerve with a cutaneous component, and the ability to separate this from the anterior branch of the axillary nerve. A hand-held nerve stimulator showed no response of the anterior branch of the axillary nerve to 0.5 milliamps, but a modest response (discernible muscle contraction, but not robust contraction and certainly not anti-gravity strength) at 2 milliamps. This prompted a reverse end-to-side nerve transfer rather than an end-to-end nerve transfer to augment recovery of her injury of the axillary nerve, based on past literature as well as our anecdotal experience with these injuries [5]. The triangular interval was opened, the radial nerve proper was identified, and a lengthy branch to the long head of the triceps was identified and dissected distally. Nerve stimulation showed a robust response to the long head of the triceps muscle. The donor nerve was divided distally and transposed proximally to enable the nerve transfer. An epineurial window within the anterior branch of the axillary nerve was created, and the reverse end-to-side nerve transfer was performed using 9-0 nylon simple interrupted sutures and reinforced with fibrin glue.

Post-nerve transfer

**Figure 1:**
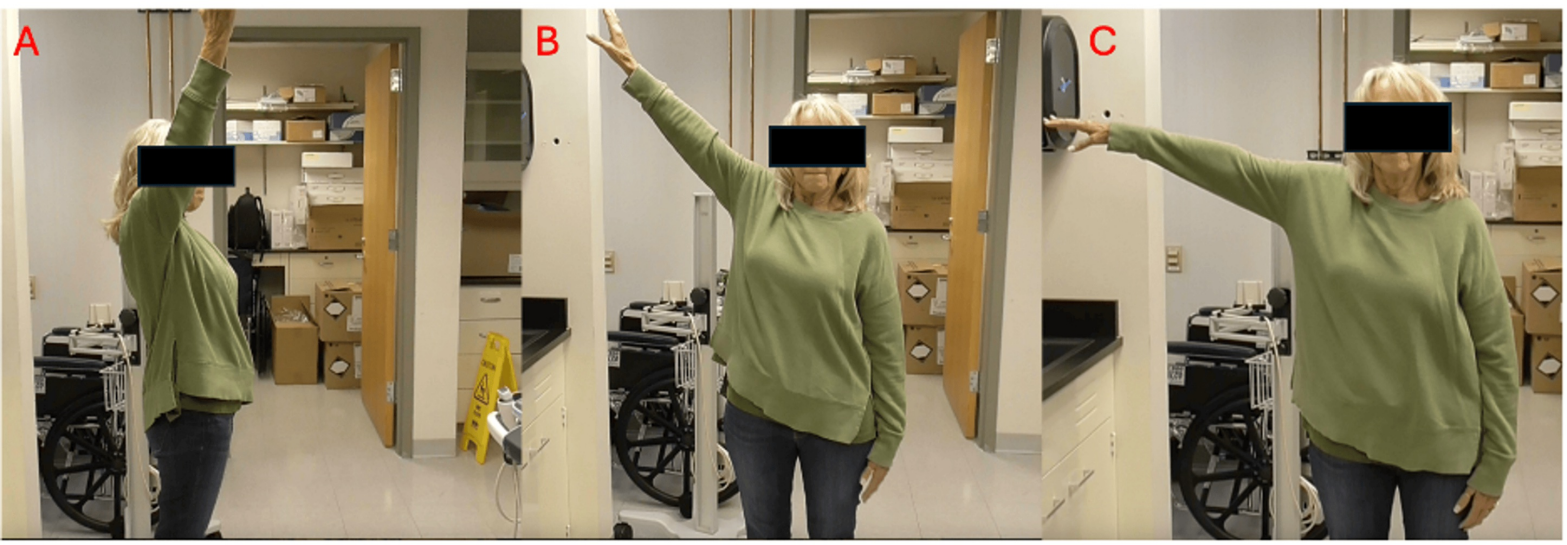
Forward Flexion, Composite Shoulder Abduction, and Abduction with Deltoid Isolated at 4 Years Postoperative

**Figure 2:**
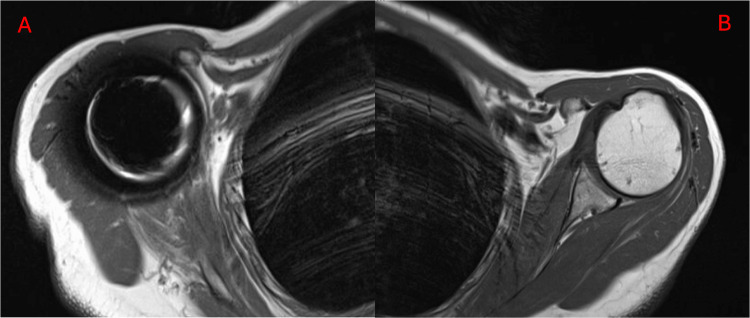
MRI at 4-year Follow-up Displaying Mild Fatty Infiltration and Atrophy of the Right Deltoid Muscle (A) Compared to Healthy Left Shoulder (B)

The patient’s initial postoperative course was uneventful, and early rehabilitation focused on mobilization of the shoulder as well as periscapular strengthening. By 2 months post-op, our patient achieved a shoulder flexion of 90° and abduction with deltoid isolation of 75°. At 7 months post-op, she was able to achieve a forward flexion of 150°, shoulder abduction of 90° with the deltoid in isolation, and a composite shoulder abduction to 160°. At four years post-op, she has improved strength, range of motion, and functional outcomes as seen in Table 3 and Figure 1. Patient-Reported Outcomes Measurement Information System (PROMIS) scores are regularly obtained at visits at our institute, and the change in scores can be seen in Table 2. Her shoulder abduction and flexion were graded at the Medical Research Council Scale (MRC) 5-, and she was able to abduct a 4 lb. dumbbell to 90° (Table 4). Bilateral shoulder MRI and US both showed mild fatty infiltration and atrophy of the right deltoid compared to the left (Figure 2). She has remained active, regularly playing racket sports and golf. Moreover, following her injury, she retired from work but has subsequently returned as a substitute school-bus driver, where she has no difficulties using her affected extremity to steer the bus.

**Table 3:** Range of Motion Values Throughout Clinical Course MRC: Medical Research Council Scale

Range of Motion	Preoperative	2 Months Postoperative	7 Months Postoperative	Final Follow-up (44 months)
Forward Flexion	60 degrees	120 degrees	150 degrees	155 degrees
Abduction with Deltoid in Isolation	NA	75 degrees	90 degrees	100 degrees
Composite Shoulder Abduction	20 degrees	160 degrees	160 degrees	160 degrees
Deltoid MRC	1	3	4-	5-

**Table 4:** Timeline Summary of Patient’s Clinical Course EMG: electromyography

Major Event	Time from Nerve Transfer Surgery
Initial Injury	4 months before
Preoperative EMG	1 month before
Forward Flexion to 90 degrees	2 months after
Near Full Range of Motion	7 months after
Near Full Strength	4 years after

## Discussion

An axillary nerve injury following rTSA can be a devastating complication given the reliance of the rTSA on deltoid function. While axillary nerve function resolves spontaneously in most patients with conservative treatment options, some axillary nerve palsies may persist, causing patients significant pain, discomfort, and shoulder dysfunction [1-3]. This case report highlights the potential treatment option of a reverse end-to-side radial-to-axillary nerve transfer for patients who show limited to no recovery after 3-4 months following their injury.

Reverse total shoulder arthroplasty (rTSA) has become an increasingly popular treatment for comminuted, multi-fragment proximal humerus fractures in the elderly; in fact, from 2010 to 2019, the frequency of rTSAs for treatment of acute proximal humerus fractures rose 1841.4% [8]. Per documentation prior to referral, our patient had no sensory deficit in the axillary nerve distribution preoperatively. No descriptive motor exams were documented prior to surgery due to pain, which aligns with our experience with these injuries. The records from the referring physician noted weakness in the right upper extremity postoperatively, but felt these findings may have been related to the preoperative block the patient received.

The axillary and suprascapular nerves are at higher risk for injury during rTSA due to their close proximity to the glenoid [9]. This is especially true for the treatment of acute proximal humerus fractures. A systematic review found that nerve injuries were most prevalent during rTSAs performed for treatment of acute proximal humerus fractures compared to other indications, and most commonly involved the axillary nerve [1]. While axillary nerve palsies often resolve spontaneously within 6-7 months, there is literature to suggest that injuries may have a poor prognosis when minimal signs of recovery are noted early on [2, 4-7]. In those situations, further surgical intervention may be considered, such as a nerve transfer, which is described in the current case.

The timing of surgical intervention in peripheral nerve palsies is difficult to predict. In situations such as this case, it is important to have a thorough, collaborative discussion with the patient regarding surgical intervention versus observation. While some literature suggests that axillary nerve palsies spontaneously resolve, others have documented their persistence, especially in the setting of a proximal humerus fracture. Gasbarro reports that only 50% of their patients with concomitant proximal humerus fracture and nerve palsy regained forward flexion to shoulder height, in addition to 50% also being unsatisfied with their result [6]. Impastato found that of patients with absent muscle recruitment at a mean 3.5 months, zero had spontaneous recovery to MRC 4 or higher following conservative treatment of their nerve palsy at an average follow-up of 731 days; moreover, of the five patients with discrete or severely reduced recruitment, only 2 of 8 muscles returned to an MRC of 4 or higher at an average follow-up of 529 days [4]. Additionally, Steenbeck observed that three of 10 patients with severe nerve injury still had continuity noted on electromyography (EMG) at 4-6 months, indicating that surgical intervention may still be indicated even when signs of reinnervation are present [7]. Given the heightened importance of the deltoid and prior literature suggesting improved deltoid recovery with quadrangular space decompression alone, the patient elected to undergo surgical exploration with possible nerve transfer [10]. Intraoperatively, there was no axillary nerve response at 0.5 milliamps and a moderate response at 2 milliamps. These intraoperative findings indicate that sole decompression of the nerve would likely not be sufficient to treat the patient’s symptoms. Schroen showed that 50% patients whose nerves were unable to be stimulated on intraoperative nerve testing had no recovery of nerve function, while those that needed more than 0.5 mA often only experienced partial recovery [11]. Based on this data and in conjunction with the preoperative EMG, a reverse end-to-side nerve transfer was elected to try to augment recovery of a presumed stretch injury of the axillary nerve [5].

Prior studies have described the performance of rTSA performed after nerve transfers to treat axillary nerve injuries. In 2017, Salazar described an rTSA after a radial-to-axillary nerve transfer and reported substantial improvements in forward flexion, external rotation, and scores on the DASH, ASES, and Simple Shoulder Test surveys [12]. Similarly, Mastracci reported a case series of three patients who underwent end-to-end axillary nerve transfers followed by subsequent rTSA [13]. Each patient, who had no range of motion preoperatively, was able to gain a minimum of 70 degrees of abduction and 95 degrees of forward flexion after both nerve transfer and rTSA. These series demonstrate the utility of nerve transfer to restore deltoid function to a level sufficient to power rTSA, but our case is differentiated by the use of nerve transfer to treat a nerve injury after rTSA. More research is needed to define the amount of deltoid strength needed for useful shoulder function in the setting of rTSA.

Following a rTSA, there is increased activation of the deltoid, indicating the relative increase in use for shoulder abduction [14]. Hence, a deficient deltoid as a result of an axillary nerve injury often leads to a poor outcome and was previously a contraindication for rTSA. However, Lädermann et al. found that of 49 patients with severe deltoid deficiency who underwent rTSA, only 9 developed postoperative complications [15]. Based on these findings, the authors concluded that while patients may not clinically perform as well, deltoid deficiency should only be considered a relative, not absolute, contraindication for rTSA. Despite these findings, our patient is an example of a satisfactory clinical outcome of rTSA in the setting of deltoid deficiency. In addition to her range of motion and strength recovery, her PROMIS score is well above the minimal clinically important difference previously established in literature [16-18]. These results suggest that nerve transfer can help optimize functionality and prognosis in patients with this challenging clinical situation.

There are several limitations to this case report. First, given that the initial injury occurred at an outside institute, we did not have complete data regarding the physical exam before the RTSA and are reliant on provider documentation outside our institution. As such, while this was likely and presumably an iatrogenic injury, we cannot definitively conclude this. We also recognize that axillary nerve palsies often spontaneously heal; however, as noted above, given reliance on the deltoid for optimal RTSA function, the patient’s preference, her lack of notable improvement, and ultimately the intraoperative nerve findings, we felt that a nerve transfer and decompression were indicated at the time. Another limitation is the fact that two procedures were performed simultaneously in the quadrangular space decompression and nerve transfer. We are unable to assess which of these procedures truly had a greater effect on the outcome; however, given the intraoperative nerve testing (lack of response at 0.5 mA following decompression), we believe the nerve transfer was responsible for more clinical benefit. Additionally, given that the patient’s rehabilitation protocol focused on periscapular strengthening, it is plausible that some of the clinical recovery could be attributed to increased strength of the rotator cuff and not solely to deltoid reinnervation. Lastly, given this is only a single case without a control group, we are unable to definitively establish treatment efficacy; however, given the returned motion/strength and overall patient satisfaction, we feel this outcome was very satisfactory.

## Conclusions

Our patient’s marked functional and symptomatic outcomes underscore the potential of nerve transfer to restore innervation, which suggests that a nerve transfer may be a viable solution for axillary nerve injuries with persistent dysfunction following rTSA. Future studies should investigate ways to measure deltoid function following triceps-to-axillary nerve transfer so that we can better counsel patients on the anticipated postoperative course and recovery following these complex procedures.
